# Influence of Monoterpenes in Biological Activities of *Nectandra megapotamica* (Spreng.) Mez Essential Oils

**DOI:** 10.3390/biom9030112

**Published:** 2019-03-21

**Authors:** Letícia J. Danielli, Tiago J.T. de Souza, Ana J. Maciel, Marco F. Ferrão, Alexandre M. Fuentefria, Miriam A. Apel

**Affiliations:** 1 Pharmaceutical Sciences Graduate Program, Federal University of Rio Grande do Sul, Av. Ipiranga, 2752, 90610 000 Porto Alegre, Brazil; letijd@gmail.com (L.J.D.); souzatjt@gmail.com (T.J.T.d.S.); anajulia.maciel@hotmail.com (A.J.M.); alexmf77@gmail.com (A.M.F.); 2 Departament of Inorganic Chemistry, Chemistry Institute, Federal University of Rio Grande do Sul, Av. Bento Gonçalves, 9500, 91501 970 Porto Alegre, Brazil; marco.ferrao@ufrgs.br

**Keywords:** biological activities, essential oil, monoterpenes, *Nectandra megapotamica*, principal component analysis, seasonal variation

## Abstract

Investigating the influence of seasonal variations on biological activities is important for pharmacological studies and metabolic engineering. Therefore, this study was conducted to determine the variation of the chemical composition of essential oils obtained from *Nectandra megapotamica* leaves, collected at different stages of plant development, as well as its influence on the biological activities. A total of 38 compounds were identified that accounted for 97–99.2% of the chemical composition of the oils. Major differences were observed in the monoterpenic fraction, representing 5.1% of the compounds identified in the productive rest phase to 37.1% in the blooming phase. Bicyclogermacrene and germacrene D were the predominant compounds identified in the oil of all collections. Furthermore, limonene, β-pinene, and spathulenol were identified predominantly in the samples of blooming and fruiting phases. The oils exhibited significant antichemotactic activity and different effects in scavenging the radical 2,2-diphenyl-1-picrylhydrazyl. Variations were also observed in the antifungal activity, with the minimum inhibitory concentrations ranging from 125 to 500 μg/mL. These results demonstrate the influence of monoterpenes, primarily limonene, α-pinene, and β-pinene, on the bioactivities of the oil. Studies investigating the variations in the chemical composition of essential oil may offer a strategy to produce a compound or a group of compounds of interest to industries with a specific pharmacological focus.

## 1. Introduction 

Essential oils are an important group of secondary plant metabolites used in large scale in cosmetics, food, and pharmaceutical industries owing to their correlated biological effects [1,2,3]. Due to their repellent or attractive properties, these metabolites have been implicated in several ecological and biological functions, and different chemical profiles have been observed throughout plant development in response to biotic or abiotic environmental factors [4]. The chemical composition of medicinal and aromatic plants and their biological effects are known to be influenced by genetic and environmental factors [5]. Such variations can be correlated with the plant collection site and the developmental phase [6] and the agronomic and environmental conditions [2], in addition to the method of extraction [7]. 

The knowledge of the changes in the chemical composition of an essential oil caused by seasonal variations is closely associated with the evaluation of its pharmacological properties and also has important applications in the area of metabolic engineering, such as in attempts to improve the yield and facilitate the accessibility of a particular compound [6]. Contributing in this context, multivariate analysis tools such as exploratory analyses using hierarchical cluster analysis (HCA) [8] and principal component analysis (PCA) [9] lead to the elucidation of those chemical structures or classes of compounds that are representative in the differentiation of samples based on the presence of the metabolites [10].

*Nectandra megapotamica* (Spreng.) Mez (Lauraceae) is popularly known in Brazil as “canela-preta,” “canela-do-mato,” or “canela-imbuia” [11]. Species of this genus have been popularly used for their antifungal, antidiarrheal, antirheumatic, and analgesic properties [12]. Previous studies on this species have reported the presence of neolignans, phenylpropanoids, lignans, and tetrahydrofurans with cytotoxic activity against leukemic cells [13], antileishmanial and antimalarial [14], trypanocidal [11], and analgesic and anti-inflammatory activities [15], confirming the properties indicated by traditional medicine. The essential oils of this plant have been reported to possess several beneficial properties such as anti-inflammatory and antitumoral activities [16] and could also neutralize the effect induced by the venom of *Bothrops diporus* [17].

A substance possessing anti-inflammatory properties, associated with antioxidant and antifungal activities, as in the case of several essential oils, is considered to be promising in the investigation of novel therapeutic agents. During infection with dermatophytes, an immune response is induced by keratinocytes [18]. This inflammatory process is fundamental to the defense of the host; however, exacerbated responses can result in tissue damage and even increase susceptibility to opportunistic pathogens [19]. In addition to this, the production of reactive species by the defense system can worsen the disease and trigger chronic inflammatory diseases, therefore indicating the importance of the combined antioxidant action of the substance [20,21].

The influence of seasonal variations on the chemical composition of essential oils is important for optimizing the production of biologically active substances. In this context, the study was conducted to evaluate the chemical composition and the antichemotactic, antioxidant, and antifungal effects of the essential oil obtained from the leaves of *N. megapotamica* collected during different developmental stages of the plant. 

## 2. Materials and Methods

### 2.1. Plant Material

Leaves of *N. megapotamica* were collected from native populations in Southern Brazil during the following phases: productive rest (March), end of productive rest (April), pre-blooming (August), blooming (August) and fruiting (December). The botanist Dr. Sérgio L. Bordignon identified the plant material, and a voucher specimen was deposited in the Herbarium of the Federal University of Rio Grande do Sul (ICN-UFRGS - 192542). This study was approved by the National Council for Scientific and Technological Development (CNPq, no. 010427/2014-7).

### 2.2. Isolation and Chemical Analysis of Essential Oil

The essential oils were obtained from fresh leaves by hydrodistillation for 4 h using a Clevenger-type apparatus. Approximately 100 g of immediately collected leaves were used for the distillation process. The yield was determined as weight/volume (*w/v*). The samples were stored at 4 °C in the dark until analysis and assessment. For chemical analysis, the essential oils were diluted in ethyl ether to a ratio of 2:100 (*v/v*). Gas chromatography–mass spectrometry (GC–MS) (Shimadzu QP5000, Kyoto, Japan) was used for analyzing the chemical composition. A capillary column of fused silica Durabond-DB5 (30 m × 0.25 mm × 0.25 μm) was used to separate the constituents. The temperature of the injector and the detector was set at 220 °C and 250 °C, respectively, and the column temperature was programmed at 60–300 °C at a rate of 3 °C/min using helium as carrier gas at a flow rate of 1 mL/min. The compounds were identified based on the comparison of retention indices calculated by linear interpolation relative to retention times of a series of n-alkanes and their mass spectra with authentic samples and with data extracted from the literature [22] or by comparison with mass spectra recorded in the database as NIST 62 and NIST 12 (National Institute of Technology and Standards, Gaithersburg, MD, USA). Relative amounts of the components were calculated based on GC peak areas.

### 2.3. Antifungal Activity

The antifungal activity of essential oils was determined against the following strains deposited in the Applied Mycology Laboratory of the Faculty of Pharmacy of the Federal University of Rio Grande do Sul: *Trichophyton rubrum* (TRU43, TRU51, TRU50, and TRU48), *T. mentagrophytes* (TME16, TME40, TME32, and TME46), *Microsporum canis* (MCA29, MCA01, MCA33, and MCA40), and *M. gypseum* (MGY50, MGY42, MGY58, and MGY42). To obtain viable cells for use in the assay, the filamentous fungi were incubated on potato dextrose agar at 32 °C for 5 days. The minimum inhibitory concentration (MIC) was determined by the broth microdilution method according to M38-A2 protocol standardized by the Clinical Laboratory Standard Institute [23]. The experiments were conducted using RPMI-MOPS culture medium (RPMI 1640 medium containing l-glutamine, without sodium bicarbonate-buffered, at pH 7.0 with 0.165 mol/L MOPS), and the oil samples were tested at a concentration range of 1.95–500 μg/mL, where 100 μL aliquots were inoculated in a flat-bottom 96-well microtiter plate. MIC was defined as the lowest concentration of the substance in which the tested microorganism did not show visible growth. The microplates were incubated at 32 °C for 72 h. The experiments were performed in triplicate using terbinafine as a positive control.

### 2.4. Radical DPPH-Scavenging Activity 

The sequestering capacity of free radicals by the oils was determined by reaction with 2,2-diphenyl-1-picrylhydrazyl (DPPH) at concentrations of 25–250 μg/mL. The samples at their respective concentrations were added to 0.004% DPPH methanolic solution and the absorbance was read in a spectrophotometer (517 nm) within 30 min of reaction. Rutin was used as positive control, and all experiments were performed in triplicate. The percentage of activity was determined by the formula %AA = (A_DPPH_ − A_sample_)/A_DPPH_ × 100 [24]. 

### 2.5. Antichemotactic Assay

The antichemotactic activity was evaluated according to the modified Boyden chamber method described by Suyenaga et al. [25]. A total of seven animals were used for this test. For obtaining rat polymorphonuclear neutrophils, 20 mL of sterile 1% glycogen (*w/v*) was injected into the peritoneum of one Wistar rat that was killed 4 h later for leukocyte collection. Before the assay, the leukocytes were treated with essential oils and positive control solubilized in Hank’s balanced salt solution (HBSS pH 7.4) at concentrations of 0.5 and 5 μg/mL for 30 min at 37 °C. A solution of neutrophils without the addition of the antichemotactic agent was used as a negative control. Plasma obtained from the blood of six rats was incubated with 65 µg/mL of lipopolysaccharide (LPS; from *Escherichia coli*) at 37 °C for 30 min and then diluted in Hank’s buffer to obtain a 20% solution (*v/v*). The treated leukocytes were added to the upper wells of the chamber, separated by a nitrocellulose filter (8.0 μm) from the chemotactic factor (LPS) present in the lower compartment. The migration of leukocytes was determined by the distance, measured in micrometers, between the filter upper plane and the lower plane containing two cells in 10 microscopic fields. Indomethacin was used as a positive control. The animal protocol was approved by and performed following guidelines established by the Institutional Animal Care and Use Committee of UFRGS [CEUA/CAr no. 25955/2014].

### 2.6. Multivariate Analysis 

The percentage areas of peaks obtained in the chromatographic analysis were tabulated and missing data were replaced by 0.001. Principal component analysis (PCA) was performed using the Chemostat software [26].

### 2.7. Statistical Analysis

Statistical analysis was performed using the GraphPad Prism 5.0 software (GraphPad Software Inc, San Diego, CA, USA) by the ANOVA method, followed by Tukey’s test, with data being expressed as mean ± standard deviation (SD). Differences were considered to be statistically significant when *p* < 0.05.

## 3. Results and Discussion

### 3.1. Chemical Composition and Yield of Essential Oils

Samples of *N. megapotamica* collected at different developmental stages were investigated in relation to yield and chemical composition of the essential oil. Data relative to climate during the collection period as well as the yield of oil are described in Table 1. The yield of the essential oil obtained from fresh leaves collected from the same individual during different periods ranged from 0.3 to 0.5%. The highest yield of oil was observed in samples collected during the austral summer (0.5%), corresponding to the productive rest phase of the plant and characterized by high temperatures and a total precipitation of 150–200 mm. The opposite result was observed for the oil obtained from samples collected in the pre-blooming stage, which presented a lower yield of 0.3%. During this period, there were lower maximum and minimum temperatures, in addition to a higher volume of total precipitation (250–300 mm). In terms of the compounds, the samples corresponding to this period (pre-blooming and blooming) exhibited higher levels of monoterpenes, with limonene being the predominant compound among the collection with a greater volume of precipitation. The differences in the yield and chemical composition of essential oils can be related to the effects of climatic and geographic factors such as temperature, ultraviolet radiation, atmospheric pollution, altitude, and water and nutrient availability, as well as the developmental stage of the plant and genetic factors [5].

Chemical analysis of the essential oils revealed considerable quantitative variations among the collections. A total of 38 compounds were identified by GC–MS, representing 97.0–99.2% of the total composition of the oils (Table 2). Sesquiterpene hydrocarbons fraction was predominant in the oils obtained from all samples (55.3–79.4%), followed by monoterpene hydrocarbons (5.1–37.1%) and oxygenated sesquiterpenes (5.3–15.2 %). Bicyclogermacrene (22.0–36.7%) and germacrene D (10.9–19.2%) were identified as the major compounds in all the oil samples. A relatively higher abundance of bicyclogermacrene was detected in the oil corresponding to samples of the productive rest (32.1% and 36.7%) and pre-blooming (33.4%) stages. Similar variations were observed for germacrene D, with values of 18.7%, 19.2%, and 16.8%, respectively, for the oils obtained from the above-mentioned phases.

The major differences were observed in the monoterpenic fraction. The quantity of this fraction increased gradually from the oils obtained from the samples of the productive rest phase to those of the blooming phase (5.1% to 37.1%, respectively), with a slight reduction in the oils obtained during the fruiting phase (20.7%). This variation was predominantly related to the quantities of α- and β-pinenes and limonene identified in the different samples. The oil extracted from the sample collected during the productive rest phase was characterized by the absence of α-pinene, the presence of β-pinene only as a minority compound (0.2%), and limonene (4.6%) as the more abundant compound in the monoterpenic fraction. In the oil corresponding to the sample collected at the end of this phase, the major compounds were α- and β-pinenes (4.3% and 6.6%, respectively), whereas there was a reduction in the abundance of limonene (2.7%). The oil obtained from the sample collected during the pre-blooming phase exhibited a predominance of limonene (14.1%), followed by β-pinene (4.1%) and α-pinene (2.7%). The opposite result was observed in the oil sample collected during the blooming phase, in which both pinenes were predominant (14.8% and 15.5%, respectively, for α- and β-pinene) and limonene constituted only 5.3%. Similar to the oils obtained from leaves collected during the productive rest and pre-blooming phases, the oil sample of the fruiting phase showed a reduced quantity of pinenes (5.0% and 6.4%, respectively) but a slight predominance of limonene (8.6%).

Regarding the hydrocarbon sesquiterpene fraction, bicyclogermacrene and germacrene D were predominant in the oils obtained from samples of all collections, with varying amounts in each (22.0–36.7% and 10.9–19.2%, respectively). In addition, β-caryophyllene (3.9–6.4%), δ-cadinene (3.9–6.8%), α-copaene (2.8–4.5%), and α-humulene (2.7–3.9%) were the major compounds with smaller variations among the oil samples. Among oxygenated sesquiterpenes, spathulenol was the major compound present in the oil obtained from all collections (1.9–3.3%) and also in the sample of the fruiting phase with a relative abundance of 9.1%. This may be related to the fact that the oil obtained from this sample showed the lowest quantity of germacrene D (10.9%) and also a reduced quantity of bicyclogermacrene (22.8%) compared with oils extracted from the other collections. Compounds of phenylpropanoid class (elemicin and (*E*)-isoelemicin) were observed in small quantities, except for the oil corresponding to the sample of the productive rest phase, which exhibited 9.4% of (*E*)-isoelemicin. According to Gobbo-Neto and Lopes [4], the collection period is one of the most important factors affecting the nature and quantity of the constituents, which are variable during the year. In addition, the developmental phase of the plant can have a considerable effect on the relative proportions of the components.

**Figure 1:**
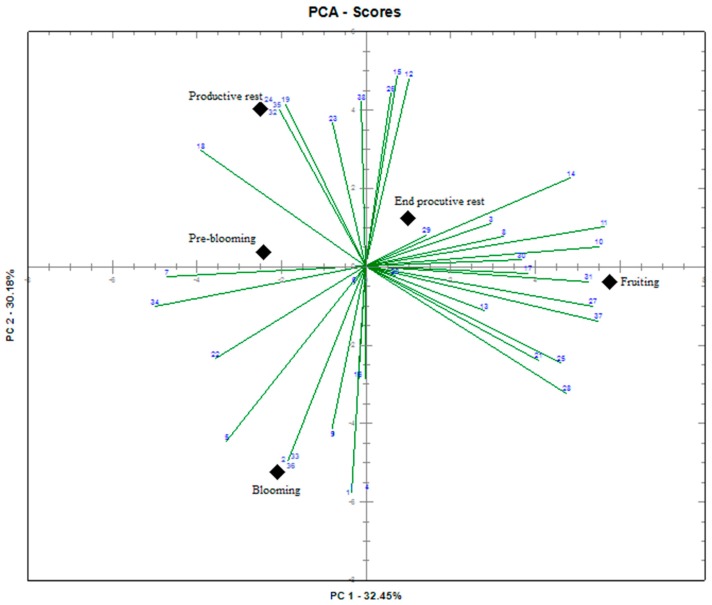
Biplot based on the first two principal components of samples of essential oils obtained from leaves of *Nectandra megapotamica* collected during different developmental phases of the plant.

To investigate possible correlations among the compounds and the collection phase due to the similarity of the chemical profile, the chemical data of the essential oils obtained from leaves collected during different stages of plant development were evaluated by PCA. The graph produced by the PCA, as shown in Figure 1, indicated different chemical profiles among the oils influenced by minor compounds. The biplot graph shows the first two components (PC1 and PC2), which together account for 62.63% of the variance. This graph distinguished two groups of plant developmental phases characterized by PC1, which corresponds to 32.45% of the total variance. This group corresponds to the oils obtained from samples of the fruiting and the end of the productive rest phases. The second group observed for the remaining PCA chart shows negative scores on the PC1 scale and corresponds to the oils obtained from samples collected in the productive rest, pre-blooming, and blooming phases. 

Furthermore, a strong correlation was observed between globulol (29), aromadendrene (14), sabinene (3), and α-copaene (8) compounds and oils obtained from the sample collected at the end of the productive rest phase, while β-elemene (11), β-cubebene (10), humulene epoxide II (31), spathulenol (27), and elemicin (37) correlated with the oil sample of the fruiting phase. In the productive rest phase, this correlation was observed primarily with cadina-1,4-diene (24), bicyclogermacrene (19), τ-cadinol (35), and 1-epi-cubenol (32). Finally, the oil extracted from the sample collected during the blooming phase correlated with camphene (2), isospathulenol (33), cubenol (36), and β-bourbonene (9) compounds.

### 3.2. Antifungal Activity

Regarding the antifungal activity, the essential oils were evaluated at concentrations of 1.95–500 μg/mL against strains of *Trichophyton* and *Microsporum* genera. MIC values in the range of 125–500 μg/mL were observed for oils obtained from samples collected during the pre-blooming, blooming, and fruiting phases (Table 3). Only one isolate of *T. rubrum* was sensitive to the oil obtained samples of the end productive rest phase at the concentration of 500 μg/mL. The oils obtained from samples of the productive rest and the end of the productive rest phases exhibited MIC values >500 μg/mL for all the tested strains, except for TRU48 that showed an MIC of 500 μg/mL.

The oils obtained from samples collected during the pre-blooming, blooming, and fruiting phases exhibited the antifungal effect for a greater number of microorganisms, with MICs ranging from 125 to 500 μg/mL. The oil from the sample of the pre-blooming phase, which contained limonene among the major compounds, showed growth inhibition of 60% of the tested strains, with MICs ranging from 250 to 500 μg/mL. A similar result was observed for the oil extracted from the sample of the fruiting phase, which inhibited 53.3% growth of the strains and exhibited an MIC of 125 μg/mL. In addition to limonene, the oil obtained from the sample of this phase contained higher amounts of spathulenol, an oxygenated sesquiterpene. The lower content of limonene and the higher contents of α- and β-pinenes in the oil extracted from the sample collected during the blooming phase correlated with a reduced antifungal effect, showing growth inhibition of only 33.3% of the strains. The presence of a higher amount of limonene in the oil obtained from the sample of the pre-blooming phase indicated a direct correlation of this compound to the inhibition of the growth of dermatophytes, considering that the oil obtained from the sample collected at the end of the productive rest phase contained only 13.8% of monoterpenic compounds and inhibited the growth of only one isolate. The lack of antifungal effect was also observed for the oil obtained from the sample collected during the productive rest phase, which contained β-pinene and limonene as the minority compounds.

In general, the antifungal activity of the essential oils is attributed to the content of oxygen-containing compounds such as oxygenated sequiterpenes [28]. This hypothesis was corroborated by the reduced MIC values (125 μg/mL) observed for the oil obtained from the sample of the fruiting phase containing spathulenol (9.1%) among the major compounds. However, Dhouioui et al. [5] reported that different samples of oil from *Aristolochia longa* L. ssp. Paucinervis, containing smaller fractions of oxygenated compounds, were more effective against the tested microorganisms, in the same manner in which α-pinene and limonene exhibited higher antifungal activity than *Wedelia prostate* total oil, with MICs ranging from 62.5 to 125 μg/mL [1]. These findings may validate the improvement in activity of the oil from obtained samples of the blooming and pre-blooming phases against those of the productive rest phase, since the former contained α- and β-pinenes and limonene as the major compounds.

### 3.3. Radical DPPH-Scavenging Activity 

The essential oils exhibited significantly lower DPPH radical-scavenging ability against rutin (positive control) at all the tested concentrations, except at 25 μg/mL (*p* < 0.05) (Table 4). The oil obtained from the sample of the pre-blooming phase exhibited a superior effect compared with oils extracted from other samples at concentrations of 150–250 μg/mL (*p* < 0.05), with the scavenging rates varying from 27.6% to 43.4%. Except at the concentration of 250 μg/mL, in which the oils obtained from the samples of the end of the productive rest, blooming, and productive rest phases exhibited antioxidant activities of 22.3%, 26.3%, and 35.9%, respectively, all the other tested concentrations exhibited an antioxidant effect of approximately or <20%.

Limonene was identified as one of the major compounds in the oil obtained from the sample of the pre-blooming phase, which demonstrated a stronger free-radical-scavenging activity. The presence of this compound in significant amounts may have contributed to its action [29]. Dai et al. [1] demonstrated that limonene was more active than α-pinene, although they found a moderate antioxidant effect for both compounds. The compounds α-pinene and β-pinene tested alone in the DPPH assay showed no free-radical-scavenging ability [30,31], indicating that the antioxidant effect observed in this study for the oils obtained from the samples of the productive rest and blooming phases can be attributed to the synergistic interaction between different compounds present in the oil. In general, for the essential oils, the stabilization of the radical DPPH effect is attributed to the presence of phenols, alcohols, and other oxygenated compounds due to their high hydrogen donation capacity [32]. Thus, (*E*)-isoelemicin, the major compound identified in the oil obtained from the sample of the productive rest phase, may have contributed to its antioxidant effect.

### 3.4. Antichemotactic Activity

**Figure 2:**
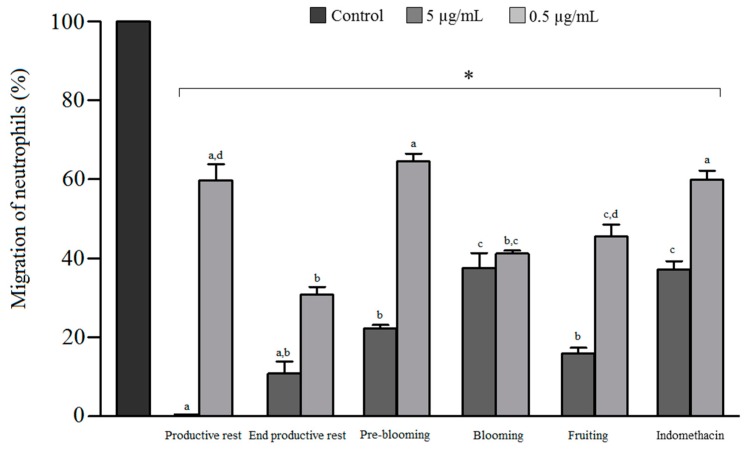
In vitro effect of the different essential oils of *Nectandra megapotamica* and indomethacin on neutrophil migration. * Significant inhibition at all tested concentrations compared with negative control. Different letters indicate a significant difference between samples at the same concentrations (*p* < 0.05) (ANOVA, followed by Tukey’s test).

The ability of essential oils to inhibit leukocyte migration was assessed by the Boyden method. All the oil samples, at the tested concentrations, exerted a statistically significant reduction in leukocyte migration compared with the negative control (*p* < 0.05), as shown in Figure 2, with the inhibition ranging from 34.6% to 100% for the samples, whereas indomethacin showed an inhibition of only 33% and 58.5%, respectively, at the concentrations of 0.5 and 5 μg/mL. Significant differences were observed among oils at the same concentration. At 5 μg/mL, the oils corresponding to the end of the productive rest, productive rest, pre-blooming, and fruiting phases exhibited a significantly higher antichemotactic effect (*p* < 0.05) than that of the oil obtained from the sample of the blooming phase, with the inhibition ranging from 77.5% to 100%. These results indicated that the presence of monoterpenes has a direct effect on the activity, because the effect increased with a decrease in the monoterpenic fraction. The oil obtained from the sample of the productive rest phase demonstrated 100% inhibition of leukocyte migration, with the monoterpenic fraction comprising only 5% of the total oil content, in addition to the predominance of the sesquiterpene hydrocarbon fraction (73.9%). On the other hand, the oil corresponding to the blooming phase exhibited a lower antichemotactic effect and a higher percentage of monoterpenes, including limonene and α- and β-pinenes as the primary compounds. 

The antichemotactic activity observed for the oil extracted from the sample of the productive rest phase can be related to the presence of (*E*)-isoelemicin, one of the major compounds, based on several reports in the literature on the anti-inflammatory activity of phenylpropanoids [33]. Studies have reported that α-pinene exerts an anti-inflammatory effect through the suppression of protein kinase and nuclear factor κ-B [34], while limonene inhibits leukocyte migration at concentrations of 1, 3, and 10 μg/mL [35]. The fact that α-pinene acts through an anti-inflammatory mechanism that is different from the one investigated in this study may explain the lower inhibitory effect of the oil obtained from the sample of the blooming phase compared with that of other oils.

In addition to being more abundant in the bloodstream among the leukocytes, neutrophils are characterized as the first defense cells recruited to the site of the injury from chemoattractant factors released by microorganisms or necrotic cells [36]. In this manner, the antichemotactic effect observed in this study indicates that the essential oils act in the acute phase of the inflammatory process. In addition to this, activation of the immune system, triggering an inflammatory process, is an important step in the defense of the host against microbial infections. However, in a contradictory manner, exacerbated inflammatory responses can cause tissue damage and even increase susceptibility to opportunistic pathogens [19]. Therefore, this property, together with the antifungal activity and the antioxidant effect observed for *N. megapotamica* samples, especially those obtained during the pre-blooming, blooming, and fruiting phases, indicates that they can act both in the healing process of one fungal infection and in relieving symptoms. During an infection, this set of biological properties contributes significantly to the rapid reduction of symptoms, the promotion of cures, and the prevention of chronicity [37].

## 4. Conclusions

This study reports differences in yield and chemical composition of essential oils obtained from the leaves of *N. megapotamica* collected during the productive rest, end of the productive rest, pre-blooming, blooming, and fruiting phases. Although the major compounds identified in the oils were common across the samples, they exhibited important quantitative differences. Such chemical variations, primarily in the content of monoterpenes, limonene, α-pinene, and β-pinene, resulted in important differences in the antichemotactic, antifungal, and DPPH radical-scavenging activities. This variability indicates the importance of qualitative and quantitative chemical characterization of *N. megapotamica* oil in relation to seasonal variations, as well as its influence on biological effects. Studies investigating the variations in the chemical composition of essential oils may offer a strategy to produce a compound or a group of compounds of interest to industries with a specific pharmacological focus.

## Figures and Tables

**Table 1:** Climatic characteristics and yield of *Nectandra megapotamica* essential oils during each collection period.

	Productive Rest	End Productive Rest	Pre-Blooming	Blooming	Fruiting
Collected period	Summer	Autumn	Winter	Winter	Spring
Total precipitation (mm)	150–200	100–150	250–300	150–200	100–150
Maximum temperature (°C)	24–26	22–24	14–18	20–22	24–26
Minimum temperature (°C)	14–16	14–16	6–10	10–12	14–16
Yield (%)	0.5	0.4	0.3	0.4	0.4

Information referring to the month of collection [27].

**Table 2:** Variability in the chemical composition of essential oils obtained from leaves of *Nectandra megapotamica* collected during different developmental phases of the plant.

ID			Compounds	RI	Relative Peak Area (%)				
					Productive Rest	End Productive Rest	Pre Blooming	Blooming	Fruiting
		**Monoterpene Hydrocarbons**							
1			α-pinene	925	-	4.3	2.7	14.8	5.0
2			Camphene	939	-	-	-	0.5	-
3			Sabinene	965	-	0.2	0.2	Tr	0.7
4			β-pinene	967	0.2	6.6	4.1	15.5	6.4
5			Myrcene	986	Tr	-	0.5	0.7	-
6			Limonene	1022	4.6	2.7	14.1	5.3	8.6
7			(*E*)-β-ocimene	1043	0.3	-	0.5	0.3	-
		**Sesquiterpene Hydrocarbons**							
8			α-copaene	1363	4.0	4.5	2.8	3.8	4.3
9			β-bourbonene	1371	0.1	Tr	0.1	0.3	0.1
10			β-cubebene	1377	0.5	0.5	0.4	0.5	0.6
11			β-elemene	1380	0.6	0.7	0.5	0.5	0.9
12			β-caryophyllene	1404	6.2	5.3	6.4	3.9	6.0
13			β-gurjunene	1414	0.1	-	-	0.1	0.2
14			Aromadendrene	1424	0.4	0.4	0.3	0.3	0.5
15			α-humulene	1437	3.9	3.8	2.8	2.7	3.2
16			Allo-aromadendrene	1444	0.2	1.0	0.2	0.9	0.3
17			γ-muurolene	1462	-	-	0.3	-	0.5
18			Germacrene D	1467	18.7	19.2	16.8	15.0	10.9
19			Bicyclogermacrene	1483	32.1	36.7	33.4	22.0	22.8
20			α-muurolene	1485	0.5	Tr	0.4	0.4	0.6
21			Germacrene A	1489	-	0.5	0.4	0.3	0.6
22			γ-cadinene	1501	0.3	Tr	0.2	0.4	0.1
23			δ-cadinene	1507	6.1	6.8	4.0	4.1	3.9
24			Cadina-1,4-diene	1513	0.2	-	-	-	-
25			Germacrene B	1537	-	-	-	0.1	0.3
		**Oxygenated** **Sesquiterpenes**							
26			(*E*)-nerolidol	1547	3.5	0.9	1.7	0.6	2.4
27			Spathulenol	1558	2.4	3.0	1.9	3.3	9.1
28			Caryophyllene oxide	1561	0.2	0.5	0.2	0.6	0.9
29			Globulol	1564	1.5	-	1.0	0.9	1.8
30			*Epi*-globulol	1571	-	1.3	0.2	0.2	0.8
31			Humulene epoxide II	1596	-	-	-	-	0.2
32			1-*epi*-cubenol	1617	0.2	-	-	-	-
33			*Iso*-spathulenol	1626	-	-	-	0.2	-
34			α-muurolol	1629	0.2	-	0.3	0.2	-
35			*τ*-cadinol	1632	0.7	-	-	-	-
36			Cubenol	1643	-	-	-	0.6	-
		**Phenylpropanoids**							
37			Elemicin	1544	0.1	0.3	-	0.2	0.7
38			(*E*)-isoelemicin	1644	9.4	-	2.6	-	4.6
		**Monoterpene hydrocarbons**			5.1	13.8	22.1	37.1	20.7
		**Sesquiterpene hydrocarbons**			73.9	79.4	69.0	55.3	55.8
		**Oxigenated sesquiterpenes**			8.7	5.7	5.3	6.6	15.2
		**Phenylpropanoids**			9.5	0.3	2.6	0.2	5.3
		**Total of compounds identified**			97.2	99.2	99.0	99.2	97.0

Compounds are listed in order of elution on DB5 column; ID: Identification of compounds represented on the graph produced by PCA (Figure 1); RI: Retention index; Tr. Traces. Percentage of peak area relative to the total area.

**Table 3:** Minimum inhibitory concentration (MIC) of essential oils from the leaves of *Nectandra megapotamica* collected during different developmental phases of the plant against dermatophytes.

Minimum Inhibitory Concentration (μg/mL)						
	Productive Rest	End Productive Rest	Pre-Blooming	Blooming	Fruiting	Terbinafine
*Trichophyton rubrum*						
TRU48	>500	500	500	250	125	0.03
TRU50	>500	>500	>500	>500	>500	0.06
TRU51	>500	>500	250	>500	500	0.008
TRU55	>500	>500	500	500	125	-
*Trichophyton mentagrophytes*						
TME16	>500	>500	500	>500	>500	0.016
TME33	>500	>500	>500	>500	>500	0.03
TME40	>500	>500	500	>500	>500	0.016
TME46	>500	>500	>500	>500	500	0.13
*Microsporum canis*						
MCA01	>500	>500	500	250	250	0.004
MCA29	>500	>500	250	>500	>500	0.008
MCA33	>500	>500	>500	>500	>500	-
MCA40	>500	>500	>500	>500	>500	1.00
*Microsporum gypseum*						
MGY42	>500	>500	500	250	125	0.016
MGY52	>500	>500	>500	>500	500	0.13
MGY58	>500	>500	500	500	125	2.00

(-) not tested.

**Table 4:** Radical DPPH-scavenging activity of essential oils from the leaves of *Nectandra megapotamica* collected during different plant development phases.

Sample	DPPH-Scavenging Activity (%)					
	250 μg/mL	200 μg/mL	150 μg/mL	100 μg/mL	50 μg/mL	25 μg/mL
Productive rest	35.9 ± 0.3 ^c^	21.7 ± 0.5 ^c^	16.3 ± 0.6 ^c^	11.4 ± 1.6 ^b^	7.8 ± 0.2 ^b^	4.1 ± 0.3 ^a^
End productive rest	22.3 ± 2.4 ^e^	9.9 ± 0.5 ^e^	6.3 ± 0.2 ^f^	4.0 ± 0.0 ^c^	0.0 ± 0.0 ^d^	0.0 ± 0.0 ^c^
Pre-blooming	43.4 ± 0.4 ^b^	37.7 ± 0.3 ^b^	27.6 ± 0.2 ^b^	10.7 ± 0.7 ^b^	5.6 ± 0.3 ^b,c^	5.0 ± 0.8 ^a^
Blooming	26.3 ± 0.2 ^d^	20.4 ± 0.2 ^c^	13.6 ± 0.3 ^d^	12.3 ± 0.3 ^b^	3.4 ± 0.3 ^c,d^	2.0 ± 0.3 ^b^
Fruiting	18.1 ± 0.2 ^f^	14.7 ± 0.2 ^d^	9.4 ± 0.2 ^e^	3.8 ± 0.4 ^c^	2.8 ± 0.4 ^c,d^	0.0 ± 0.0 ^c^
Rutin	96.1 ± 0.3 ^a^	93.5 ± 0.7 ^a^	90.5 ± 0.6 ^a^	50.8 ± 3.9 ^a^	13.2 ± 2.6 ^a^	2.3 ± 0.4 ^b^

Rutin, positive control. Different letters indicate a significant difference between samples in the same concentrations (*p* < 0.05) (ANOVA followed by Tukey’s test).
